# SEOM clinical guidelines for anaemia treatment in cancer patients (2020)

**DOI:** 10.1007/s12094-021-02580-2

**Published:** 2021-03-25

**Authors:** Y. Escobar Álvarez, R. de las Peñas Bataller, J. Perez Altozano, S. Ros Martínez, A. Sabino Álvarez, A. Blasco Cordellat, E. Brozos Vázquez, J. Corral Jaime, I. García Escobar, C. Beato Zambrano

**Affiliations:** 1 Department of Medical Oncology, Hospital General Universitario Gregorio Marañón, Madrid, Spain; 2 Department of Medical Oncology, Consorcio Hospitalario Provincial de Castellón, Castellón de la Plana, Spain; 3 Department of Medical Oncology, Hospital Virgen de Los Lirios, Alcoy, Spain; 4 Department of Medical Oncology, Hospital Universitario Virgen de la Arrixaca, Murcia, Spain; 5 Department of Medical Oncology, Hospital Universitario Puerta del Mar, Cádiz, Spain; 6 Department of Medical Oncology, Consorcio Hospital General Universitario, Valencia, Spain; 7 Department of Medical Oncology, Hospitalario Clínico Universitario de Santiago, la Coruña, Spain; 8 Department of Medical Oncology, Clínica Universidad de Navarra, Madrid, Spain; 9 Present Address: Hospital General Universitario Virgen de las Nieves, Granada, Spain; 10 Department of Medical Oncology, Hospital Universitario de Jerez de la Frontera, Cádiz, Spain

**Keywords:** Anaemia, Erythropoiesis stimulating agents, Iron supplements, Transfusion of blood products

## Abstract

Anaemia is defined by the presence of haemoglobin (Hb) levels < 13 g/dL in men and 12 g/dL in women. Up to 39% of cancer patients present it at the time of diagnosis and up to 40% have iron deficiency. Anaemia causes fatigue, functional deterioration and a reduction in the quality of life; it has also been associated with a poorer response to anti-tumour treatment and lower survival. Basic diagnostic tests for anaemia are simple and should be a routine part of clinical practice. These guidelines review the available evidence on the use of different therapies for treating anaemia: erythropoiesis-stimulating agents, iron supplements, and transfusion of blood products.

## Introduction

Anaemia is defined by the presence of haemoglobin (Hgb) levels < 13 g/dl in men and 12 g/dl in women. Iron deficiency (ID) is characterized by low transferrin saturation (TSI < 20%) and can be absolute (depleted iron stores, serum ferritin < 30 ng/ml) or functional (normal or increased serum ferritin); both are common complications in patients with solid tumours.

Up to 39% of cancer patients present anaemia at the time of diagnosis and up to 40% present ID; of these, about a third have Hgb levels < 12 g/dl. In addition, up to 53% of patients who do not present anaemia at diagnosis will develop it during chemotherapy (CT) and/or radiotherapy (RT) treatment, so that up to 67% of cancer patients will present anaemia at some time during the evolution of their disease [1].

Anaemia causes fatigue, functional deterioration and reduced quality of life [2]. It has also been associated with a worse response to anti-tumour therapy and shorter survival.

The aetiology of cancer-related anaemia is multifactorial, and two or more factors can be present in the same patient. For example, anaemia may be caused by a combination of:

- A direct effect of the cancer (tumour bleeding, invasion of the bone marrow).
- Chemical factors produced by the tumour (auto-antibodies, inflammatory cytokines that affect erythropoietin production and block iron metabolism).
- An effect of cancer therapy (cell death induced by CT, RT, tyrosine kinase inhibitors (TKI) and monoclonal antibodies (mAb).

Drug-induced anaemia is graded according to the CTCAE [3] (Table 1).

**Table 1:** Anaemia grades according to the Common Terminology Criteria for Adverse Events (CTCAE) v 5.0 published on 27 November 2017

Grade 1	Grade 2	Grade 3	Grade 4	Grade 5
(Hgb) < LLN—10 g/dl; < LLN—6.2 mmol/l, < LLN—100 g/l	Hgb < 10.0–8.0 g/dl; < 6.2–4.9 mmol/l; < 100–80 g/l	< 8.0 g/dl	Potentially life-threatening consequences Urgent intervention indicated	Death
		< 4.9 mmol/l		
		< 80 g/l		
		Transfusion indicated		

*LLN* lower limit of normality

Cancer-related anaemia, therefore, is a multifactorial problem with immunologic, nutritional and metabolic components that affect its severity. For this reason, reversible causes must be identified and the different therapeutic options available for treatment must be used correctly (level of evidence I, recommendation grade A).

## Algorithm for the diagnosis of anaemia in cancer patients

A correct diagnosis of anaemia requires a medical history that includes: personal history, status of the oncological disease (the type of primary tumour and stage of the disease, the different treatments received, chemotherapies with a high risk of haematological toxicity and their dates of administration are the most important data), clinical history and complete physical examination.

The most common symptoms presented by patients with anaemia are fatigue, dyspnoea on exertion, oedema in the lower limbs, cognitive impairment, confusion and depression. Clinical signs include pallor, tachycardia, and worsening of performance status (PS-ECOG) [5].

Blood tests required include a basic blood count, a ferrokinetic study (serum ferritin levels and transferrin saturation), biochemistry with creatinine (to assess kidney function), and C-reactive protein (marker of chronic inflammation) [5].

**Fig. 1:**
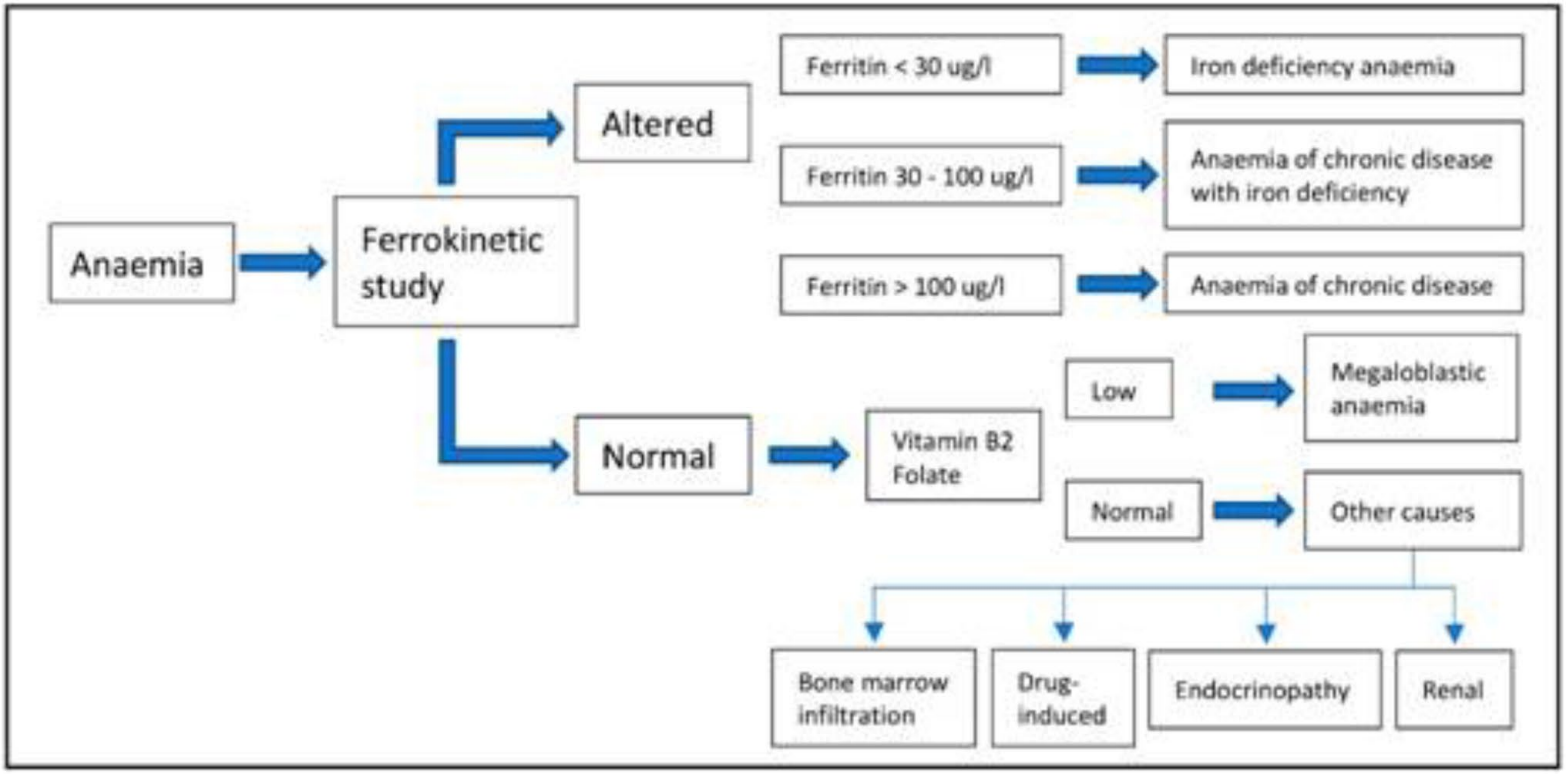
Algorithm for the diagnosis of anaemia in cancer patients

Since anaemia in cancer patients can be multifactorial, other complementary tests should be requested to determine the cause: occult blood in stool, urinary sediment, blood smear, coagulation tests, thyroid hormones and immunological studies [6]; bone marrow biopsy if tumour infiltration is suspected, an imaging test, such as computerized tomography and gastrointestinal endoscopy, to identify possible bleeding can also be useful. The algorithm shown in Fig. 1 can be used to diagnose anaemia in cancer patients.

It is important to differentiate between iron deficiency anaemia and chronic cancer-induced anaemia. Table 2 shows the parameters used to differentiate between these entities.

**Table 2:** Differences between iron deficiency anaemia and cancer-induced anaemia. (Adapted from Dr Muñoz Langa) [2]

	Iron deficiency anaemia	Cancer-induced anaemia
Reticulocytes	High or low	Low
MCV/MCH*	Low or normal	Low or normal
Serum iron	Low	Low or normal
Serum ferritin	Low	Normal or high
Transferrin Saturation Index	Low or normal	Low
Total binding capacity	High	Low
Serum transferrin receptor	High	Normal
Bone marrow iron deposits	Absent	Present

*MCH mean corpuscular haemoglobin, *MCV* mean corpuscular volume

## Treatment of anaemia in cancer patients

### Erythropoyetin-stimulating agents (ESA)

The cloning of the human erythropoietin (EPO) gene in 1984 opened a new option of treatment for cancer-related anaemia. Numerous clinical trials have shown the efficacy and safety equivalence of different ESAs, which reduce transfusion needs in patients with chemotherapy-induced anaemia, achieve more sustained correction of anaemia, are associated with fewer risks than transfusions, and improve both blood flow and quality of life [7, 8].

However, the use of these agents has decreased considerably since 2005 following the publication of data that associated them with decreased survival.

Below, we analyse the indications and risks of the different ESAs available, as shown in Table 3.

**Table 3:** ESAs in cancer patients: indications, risks and recommendations [10, 15–17]

Drug	Dose	Indications	Risks	Recommendations
Epoetin alfa (EPREX/ERYPO, BINOCRIT, ABSEAMED, HEXAL)	SC injection: 150 IU/kg (10,000 IU) 3 times a week (Level of evidence III, grade of recommendation C) or 450 IU/kg (40,000 IU) once a week (Level of evidence I, GRADE OF RECOMMENDATION A)	To treat anaemia and reduce the need for blood transfusion in adult patients receiving chemotherapy for the treatment of solid tumours, malignant lymphoma or multiple myeloma, and at risk of transfusion as assessed by the patient’s general status	Common: VTE High blood pressure Uncommon: Allergic reaction Thrombocytopaenia Pure red cell aplasia	Its use is recommended solely in cases of symptomatic anaemia as an alternative to transfusion, in patients with a positive benefit/risk balance In patients who are not candidates, an increased risk of VTE, tumour progression, and reduced survival have been observed
Epoetin beta (NEORECORMON)	SC injection: 450 IU/kg (30,000 IU) once a week (Level of evidence I, grade of recommendation A)	* To treat symptomatic anaemia in adult patients with solid tumours treated with chemotherapy *To treat anaemia in adult patients with multiple myeloma, low-grade non-Hodgkin lymphoma, or chronic lymphatic leukaemia who present relative erythropoietin deficiency (inadequate serum levels for the degree of anaemia) and are receiving anti-cancer therapy	Common: VTE High blood pressure Uncommon: Allergic reaction Thrombocytopaenia Pure red cell aplasia	Its use is recommended solely in cases of symptomatic anaemia as an alternative to transfusion, in patients with a positive benefit/risk balance In patients who are not candidates, an increased risk of ETV, tumour progression, and reduced survival have been observed
Epoetin theta (EPORATIO, BIOPOIN)	SC injection: 20,000 IU weekly, which can be doubled in 4 weeks if Hgb does not increase by at least 1 g/dl (Level of evidence I, grade of recommendation A)	Symptomatic anaemia in non-myeloid cancer patients treated with chemotherapy	Common: VTE High blood pressure Uncommon: Allergic reaction Thrombocytopaenia Pure red cell aplasia	Its use is recommended solely in cases of symptomatic anaemia as an alternative to transfusion, in patients with a positive benefit/risk balance In patients who are not candidates, an increased risk of ETV, tumour progression, and reduced survival have been observed
Epoetin zeta (RETACRIT, SILAPO)	SC injection: 150 IU/kg (10,000 IU) 3 times a week or 450 IU/kg (40,000 IU) weekly (Level of evidence I, grade of recommendation A)	To treat anaemia and reduce the need for blood transfusion in adult patients receiving chemotherapy for the treatment of solid tumours, malignant lymphoma or multiple myeloma, and at risk of transfusion as assessed by the patient’s general status	Common: VTE High blood pressure Uncommon: Allergic reaction Thrombocytopaenia Pure red cell aplasia	Its use is recommended solely in cases of symptomatic anaemia as an alternative to transfusion, in patients with a positive benefit/risk balance In patients who are not candidates, an increased risk of ETV, tumour progression, and reduced survival have been observed
Darbepoetin alfa (ARANESP)	SC injection: 2.25 µ/kg weekly or 6.75 µ/kg every 3 weeks (Level of evidence I, grade of recommendation A)	To treat symptomatic anaemia in adult patients with non-myeloid tumours treated with chemotherapy	Common: VTE High blood pressure Uncommon: Allergic reaction Thrombocytopaenia Pure red cell aplasia	Its use is recommended solely in cases of symptomatic anaemia as an alternative to transfusion, in patients with a positive benefit/risk balance In patients who are not candidates, an increased risk of ETV, tumour progression, and reduced survival have been observed

#### Indications for ESAs

Leading agencies (EMA, FDA) and medical societies (ASCO/ASH, ESMO, NCCN) recommend the use of ESAs in [9, 10]:

- Patients with solid tumours and symptomatic anaemia under treatment with chemotherapy (level of evidence I, grade of recommendation A) or chemoradiotherapy (level of evidence II, grade of recommendation B).
- Patients with Hgb levels < 10 g/dl, or asymptomatic anaemia with Hgb levels < 8 g/dl after correction of iron levels and other underlying causes (level of evidence I, grade of recommendation A).
- In patients with Hgb < 7–8 g/dl and/or symptomatic anaemia, red blood cell transfusion should be considered before ESAs (level of evidence II, grade of recommendation B).

#### Risks and complications

Venous thromboembolism (VTE): In 2005, the results of some studies that linked the use of ESAs with an increase in mortality in cancer patients caused some alarm. Subsequent analyses found that this effect was limited to patients treated with Hgb levels > 12 g/dl [11, 12]; therefore, prophylactic treatment in non-anaemic patients is not advised (level of evidence I, grade of recommendation A) due to the increased risk of thromboembolic complications. In these cases, other options should be considered, or the risks should be evaluated individually in elderly patients, patients on bed rest, with heart failure, thrombocytosis, a history of VTE, adenocarcinoma (particularly some subtypes, or pancreatic cancer) and with some cancer treatments. The use of antithrombotic prophylaxis or aspirin is not recommended in these cases [12, 13].

Other less common complications are [7]: Pure red cell aplasia (described in patients with chronic renal failure and caused by the appearance of anti-erythropoietin antibodies), arterial hypertension, thrombocytopaenia and allergic reactions.

#### Controversy: survival and disease control

The lower survival observed in cancer patients with anaemia appears to be associated with a poorer response to certain therapies when tissue hypoxia is present. Based on this finding, some authors have suggested that achieving Hgb > 12 g/dl with ESAs will improve survival in patients receiving chemoradiotherapy [11, 14]. Subsequent studies did not confirm this hypothesis, and suggested that this might be due to the appearance of thromboembolic phenomena in patients with Hgb 13–16 g/dl due to the presence of erythropoietin receptors on the surface of tumour cells that would promote angiogenesis, tumour growth and therapeutic failure, and also to the activation of neovascularization by mechanisms independent of the erythropoietin receptor.

After analysing these studies, and on the assumption that ESAs are used in patients with symptomatic anaemia and Hgb < 10 g/dl, various agencies and scientific societies have maintained their recommendation for use of ESAs, adding that they should be avoided in patients receiving therapy for curative intent and in patients with advanced tumours but long-term survival expectations, even if they develop anaemia secondary to treatment [11, 12]. (Level of evidence IV, grade of recommendation C).

Table 3 shows the different ESAs available with their indications, risks and recommendations for use.

### Oral and intravenous iron supplementation

The ECAS study found that more than 40% of patients included had iron deficiency (ID) and, of these, approximately one-third had Hgb levels < 12 g/dl; the same study indicated that the prevalence of ID in patients with solid tumours was higher than in haematological cancers and that, in these solid tumours, the prevalence of ID correlated with a more advanced tumour stage at the time of diagnosis, worse tumour response to treatment with chemotherapy and/or radiotherapy, and worse clinical status [18].

#### Indications for iron supplementation

Iron supplementation should be considered in patients undergoing chemotherapy who have anaemia with Hgb ≤ 11 g/dl or Hgb decrease ≥ 2 g/dl from a baseline level ≤ 12 g/dl [19].

It is important to note that ID can also be associated with impaired physical function, weakness and fatigue even in the absence of anaemia, so iron supplementation must also be considered in this circumstance [20].

The following situations are established based on three laboratory parameters to be determined before and during cancer therapy (serum iron—SI, transferrin saturation index — TSI, and ferritin) (level of evidence II and grade of recommendation A) [21]:

*Possible functional iron deficiency* (ferritin 500-800 ng/ml and TSI > 50%). In this case, treatment with an erythropoietin stimulating agent (ESA) or with iron supplements is not recommended; in certain selected patients, the use of intravenous (IV) iron may be considered to avoid the need for transfusion. All iron supplementation should be suspended when ferritin > 800 ng/dl and TSI > 50%.

*Functional iron deficiency* (ferritin between 30-500 ng/ml, TSI < 50% and SI <30 µ/dl). This shows insufficient availability of iron despite adequate iron stores. Administration of IV iron together with ESAs is recommended (unless the latter are not indicated), as this improves the haematological response and reduces the number of transfusions. There is insufficient clinical evidence to indicate the routine use of IV iron in monotherapy without the concomitant use of ESAs.

*Absolute iron deficiency* (ferritin < 30 ng/ml, TSI < 20% and SI < 30 µ/dl). Indicates that iron stores are depleted. Oral or intravenous iron supplements can be given. Oral iron should only be administered in the absence of inflammation; if no response is obtained after four weeks, switch to IV iron.

#### Risks of iron supplementation

Although long-term side effects have not been fully established, IV iron therapy does not increase the risk of infections, thromboembolic events, or cardiovascular disease; however, it should be avoided in patients with active infection or concomitant cytotoxic chemotherapy; specifically, IV iron should be given before or after chemotherapy or at the end of a treatment cycle (level of evidence III and grade of recommendation C) [19]. Like any other drug, iron can induce allergic reactions [22]. There is no clinical evidence linking IV iron therapy to cancer development or progression; in research models evaluating iron as a possible promoter of tumour growth, this effect has not been demonstrated [2, 3].

#### Iron supplement presentations

Iron can be administered orally or intravenously. Although the oral form is suitable for most patients with iron deficiency anaemia, many with anaemia secondary to chemotherapy do not respond, may be intolerant, or may require higher doses than can be achieved with oral supplementation. In these cases, intravenous therapy is a better option [24]. Several studies in which iron supplementation was given in conjunction with ESAs suggest that intravenous iron is superior to oral iron in improving haemoglobin response rates [25, 26]. No studies on iron supplementation in conjunction with ESAs provide evidence on how or when to repeat iron administration after the maximum initial dose has been administered.

The most common adverse events associated with intravenous iron are: hypotension, hypertension, nausea, vomiting, diarrhoea, pain, fever, dyspnoea, itching, headache, and dizziness [27].

The dosages of different intravenous iron supplements currently available are described in Table 4.

**Table 4:** Iron compounds and approved dosages in patients with solid tumours and haematologic malignancies

Compound	Iron sucrose	Low molecular weight dextran iron	Ferric carboxymaltose iron
Test dose	Medical judgement, depending on the risk of reaction	Slow infusion of 25 mg over 15 min	Medical judgement, depending on the risk of reaction
Dosage	200 mg IV over 30–60 min (every 2–3 weeks) or 200 mg IV over 2–5 min, 5 times in 14 days Individual doses > 300 mg not recommended Total dose = 1000 mg	100–200 mg IV over 30 min Repeat dosing once a week for 10 doses for a total of 1000 mg or Full dose infusion over 4–6 h (total dose calculated in 500 ml 0.9% NaCl solution at 175 ml/h)	750 mg IV in patients ≥ 50 kg and repeat dose at least once 7 days later or 15–20 mg/kg IV in patients < 50 kg and repeat dose at least once 7 days later Minimum infusion time: 15 min Total dose = 1500 mg

### Treatment of cancer-induced anaemia by transfusion of erythrocytary derivatives

Data on red blood cell (RBC) transfusions in cancer patients come mainly from studies in surgical patients. Studies performed in large population groups and meta-analyses suggest an independent association between RBC transfusion and an increased risk of mortality, morbidity and cancer recurrence [28–31]. However, there are few randomized studies in patients undergoing chemotherapy [32, 33].

Standard RBCs are obtained by fractionating whole blood. Currently, nearly all blood is fractionated by centrifugation into its main components: red blood cells, plasma and platelets. RBCs are stored suspended in a conservation medium containing an anticoagulant (citrate) together with glucose, adenine and phosphate, intended to maintain ATP levels by means of glycolytic metabolism. RBCs are stored at 4° ± 2° C to reduce their metabolic requirements and thus prolong conservation and delay bacterial growth in the rare event of the bag being accidentally contaminated by bacteria.

RBC transfusion in cancer patients is indicated above all in patients with severe anaemia and symptoms that require rapid recovery of haemoglobin and haematocrit levels. Elevation of these parameters and the corresponding symptomatic improvement will be transitory; therefore, the aetiology of anaemia should always be investigated.

In patients with no cardiovascular risk factors, transfusion is indicated if Hgb < 7–8 g/dl (Htc: 21–24%) [34–36] and very rarely if Hgb > 9–10 g/dl (Htc: 27–30%). Unless justified, Hgb levels should not be allowed to remain below 7–8 g/d for any length of time. In patients with cardiovascular risk factors, particularly coronary heart disease, the minimum Hgb threshold should be higher, around 9 g/d [37]; patients with symptomatic anaemia should be transfused, regardless of these Hgb thresholds.

The correct RBC dose is calculated considering that 1 unit RBC increases Hgb from 1 to 1.5 g/l and haematocrit from 2 to 3%; therefore, dosage must be titrated individually, considering the characteristics of the patient and the minimum volume needed to correct the symptoms.

Immunosuppressed patients, due to the risk of post-transfusion graft-versus-host disease (GVHD), should receive irradiated blood components. Washed RBCs are indicated in patients with a history of severe post-transfusion allergic or anaphylactic reactions.

The main drawbacks of blood transfusion include the increased risk of thrombotic events and transfusion-related adverse effects. These include immediate reactions (during transfusion or over the following 24 h), such as acute haemolytic reaction, allergic reactions, transfusion-related acute lung injury (TRALY), volume overload, non-immune haemolytic anaemia, hypotensive reactions, and fever; and delayed reactions, such as delayed haemolytic transfusion reaction, transfusion-related graft-versus-host disease, transmission of infectious diseases, transfusion hemosiderosis, and particularly common in cancer patients, allergic reactions and volume overload.

## Conclusion

According to the above information, the following recommendations are established for anaemia treatment in cancer patients:

### Recommendations for ESA administration

#### Indications

Patients with solid tumours and symptomatic anaemia under treatment with chemotherapy (level of evidence I, grade of recommendation A) or chemoradiotherapy (level of evidence II, grade of recommendation B) who present Hgb levels < 10 g/dl or asymptomatic anaemia with Hgb levels < 8 g/dl, after correction of iron levels or other underlying causes (level of evidence I, grade of recommendation A).

ESAs should not be used in patients who are not receiving chemotherapy (level of evidence I, grade of recommendation A).

#### Duration and dosage

Administer until stable Hgb values that avoid or reduce the need for red blood cell transfusion have been achieved, without exceeding 12 g/dl (level of evidence IV, grade of recommendation B).

Increasing the dose or switching drugs after 6–8 weeks of treatment in non-responders is not recommended, except in the case of epoetin theta; instead, treatment should be suspended (level of evidence II, grade of recommendation B).

#### Toxicity and contraindications

The risk of thromboembolic events must be carefully evaluated and the patient duly informed. ESAs should not be used in patients with poorly controlled hypertension. (Level of evidence I, grade of recommendation A).

### Recommendations for iron supplementation

#### Indications

Iron supplementation should be considered in patients undergoing chemotherapy who have anaemia with Hgb ≤ 11 g/dl or Hgb decrease ≥ 2 g/dl from a baseline level ≤ 12 g/dl.

IV iron + ESA is recommended to treat functional iron deficiency (ferritin 30–500 ng/ml, TSI < 50%, serum Fe < 30 µ/dl) (level of evidence II, grade of recommendation A).

Oral or intravenous iron is recommended to treat absolute iron deficiency (ferritin < 30 ng/ml, TSI < 20%, serum Fe < 30 µ/dl). If no response is obtained with oral treatment after four weeks, switch to IV iron. (Level of evidence II, grade of recommendation A).

Neither ESA nor iron supplementation is recommended to treat possible functional iron deficiency (ferritin 500–800 ng/ml and TSI > 50%) All iron supplementation should be suspended when ferritin > 800 ng/dl and TSI > 50%. (Level of evidence II, grade of recommendation A).

### Toxicity and contraindications

Iron does not increase the risk of infections, thromboembolic events, or cardiovascular morbidity; IV iron should be administered before or after chemotherapy or at the end of a treatment cycle (level of evidence III, grade of recommendation C). There is no clinical evidence linking IV iron therapy to cancer development or progression.

### Recommendations for blood transfusion

Consider red blood cell transfusion in patients with Hb < 7–8 g/dl (and < 9 g/dl if cardiovascular risk factors are present) and/or severe symptoms of anaemia that need rapid correction of Hgb or symptoms (level of evidence II, grade of recommendation B).
